# AI-Assisted Fracture Detection in Orthopedic and Trauma Imaging: Where It Works, Where It Fails, and Principles for Safe Clinical Deployment

**DOI:** 10.3390/diagnostics16101420

**Published:** 2026-05-07

**Authors:** Wojciech Michał Glinkowski, Paweł Kamiński, Rafał Obuchowicz

**Affiliations:** 1 Center of Excellence “TeleOrto” for Telediagnostics and Treatment of Disorders and Injuries of the Locomotor System, Department of Medical Informatics and Telemedicine, Medical University of Warsaw, 02-091 Warsaw, Poland; w.glinkowski@gmail.com; 2 Clinic of Locomotor Disorders, Andrzej Frycz Modrzewski University, 30-705 Krakow, Poland; pawelkam@mp.pl; 3 Malopolan Orthopedic and Rehabilitation Hospital, Aleja Modrzewiowa 22, 30-224 Krakow, Poland; 4 Department of Diagnostic Imaging, Jagiellonian University Medical College, 31-008 Krakow, Poland

**Keywords:** artificial intelligence, fracture detection, musculoskeletal imaging, diagnostic accuracy, clinical governance, risk stratification, implementation science, second-reader systems, emergency radiology, orthopedic trauma

## Abstract

Background: Missed fractures on initial imaging assessments remain a clinically significant source of diagnostic errors in orthopedic and trauma care. AI-assisted imaging tools are increasingly integrated into fracture detection workflows. However, their diagnostic benefits and safety vary substantially across anatomical regions, clinical contexts, and levels of reader experience. Purpose: To synthesize the current evidence on the diagnostic impact of AI-assisted fracture detection and to discuss evidence-informed principles for safe and selective clinical deployment. Methods: A structured narrative synthesis of meta-analyses, multi-reader, multi-case observer studies, and real-world implementation investigations was performed. Diagnostic performance patterns were examined across anatomical regions and levels of reader experience. No quantitative pooling or reanalysis of the primary data was performed. The findings were synthesized across anatomical regions, reader-experience groups, and implementation-relevant clinical contexts. Results: Across studies, AI-assisted interpretation was generally associated with moderate gains in sensitivity and lower missed-fracture rates compared with unaided human reading, while largely preserving specificity. The diagnostic benefit was greatest among less-experienced readers in high-volume emergency settings. Performance was strongly anatomy-dependent: consistent and clinically meaningful improvements were observed for hip and appendicular skeleton fractures; intermediate benefits with increased false-positive burden were reported for wrist and rib fractures; and inferior sensitivity relative to expert interpretation was documented for cervical and vertebral spine injuries. Conclusions: AI-assisted fracture detection improves diagnostic safety when implemented as a structured second-reader tool; however, its effectiveness depends heavily on anatomy. Available evidence supports selective, risk-stratified deployment, guided by anatomy-specific risk considerations and supervised clinical use, rather than indiscriminate or autonomous use, to maximize benefits and minimize patient safety risks in orthopedic and trauma imaging.

## 1. Introduction

Missed fractures during the initial imaging assessment remain a significant and clinically consequential challenge in orthopedics and trauma care [1,2]. Undetected injuries can lead to delayed or inappropriate treatment, prolonged morbidity, and increased medicolegal risk, particularly in high-throughput emergency settings, where time pressure, variable reader expertise, and subtle imaging findings increase the likelihood of diagnostic oversight [3,4,5,6,7,8].

Recent advances in artificial intelligence (AI), particularly deep learning-based image analysis, have accelerated the development of fracture detection tools, most commonly for plain radiographs [9,10,11,12]. Several commercial systems have been approved for clinical use, and early studies suggest that AI assistance can improve fracture detection when used to support, rather than replace, human readers, typically as a structured second reader or decision-support system [13,14,15,16,17,18,19,20,21,22,23,24,25,26,27,28,29].

Recent systematic reviews and meta-analyses suggest that stand-alone AI systems can achieve high diagnostic accuracy in radiographic fracture detection, in some settings, approaching expert-level performance and often exceeding that of non-specialist readers [11,21,30,31,32].

However, this benefit is inconsistent across clinical contexts. AI assistance appears to provide the most consistent and clinically meaningful gains when used as an assistive second reader, particularly for hip and appendicular skeletal fractures and among less-experienced readers in emergency settings [10,21,33,34,35,36].

In contrast, the performance is weaker and more variable in anatomically complex or subtle injury patterns, including cervical and vertebral spine fractures, multiple fractures, and selected avulsion injuries [10,36,37,38,39,40]. Recent reviews have summarized overall diagnostic accuracy well, whereas reader-experience effects, anatomy-specific variation, and implementation-relevant clinical considerations have been addressed less consistently across the literature [11,21,41].

The key clinical question is not merely whether AI can detect fractures, but rather where, in whom, and under what conditions it can reduce the rate of missed injuries without introducing unacceptable diagnostic risks. Accordingly, this review aims to: summarize reported diagnostic patterns of AI-assisted fracture detection, examine how these patterns vary by anatomical region and reader experience, and discuss selected considerations relevant to supervised clinical implementation. The primary focus of this review is fracture detection on plain radiographs, where AI is most commonly proposed as a second-reader or decision-support tool in initial trauma assessment. Additional non-radiographic evidence is considered only where directly relevant to reference-standard interpretation or clinically indicated escalation of diagnostic work-up.

## 2. Materials and Methods

### 2.1. Study Design

This study was designed as a structured narrative review of AI–assisted fracture detection in orthopedic and trauma imaging, with a primary focus on radiograph-based diagnostic support. A formal systematic review or meta-analysis was not performed because the available literature is highly heterogeneous with respect to anatomical targets, clinical settings, reader populations, AI tools, reference standards, and reported outcome measures. Accordingly, a structured narrative approach was used to identify clinically relevant patterns in diagnostic performance, reader-experience-dependent effects, anatomy-specific variation, and implementation-related risk. Additional non-radiographic studies were considered only when directly relevant to reference-standard interpretation in spinal fracture research or to clinically indicated escalation imaging for occult fractures. The core review set was defined by the structured search strategy and eligibility criteria described below. Earlier or non-core references were cited selectively only for contextual framing, methodological background, or clinically relevant interpretation and were not treated as part of the core synthesis dataset.

### 2.2. Literature Search

A literature search was conducted in PubMed, Web of Science Core Collection, Scopus, ScienceDirect, and Google Scholar to identify studies that evaluated AI-assisted fracture detection on plain radiographs in clinically relevant musculoskeletal imaging settings. The searches were restricted to articles published from 1 January 2021, to 31 March 2026, and were limited to English-language articles and reviews. An initial exploratory PubMed search using a core combination of terms (“artificial intelligence” AND fracture AND x-ray/radiograph, Title/Abstract) yielded 178 records. A more clinically focused PubMed strategy incorporating diagnostic accuracy and implementation terms (e.g., sensitivity, specificity, observer, multi-reader, reader performance, implementation, workflow, decision support, and second reader) identified 325 records prior to screening and deduplication. Parallel searches with analogous clinically focused strategies were performed in the Web of Science Core Collection, Scopus, and ScienceDirect databases. Only articles reporting fracture-level diagnostic outcomes on plain radiographs were retained after manual screening. Google Scholar was used only as a supplementary tool for citation chasing and identifying recent or ahead-of-print articles, not as a primary search platform. The purpose of the search was to identify representative and methodologically informative evidence for structured narrative synthesis rather than to generate an exhaustive dataset for a quantitative pooling analysis. These searches were intended to identify a clinically informative core review set rather than an exhaustive corpus for formal quantitative synthesis. The full database-specific search strategies, including field restrictions and limits, are provided in Supplementary Table S1.

### 2.3. Eligibility Criteria and Study Selection

Studies were eligible if they evaluated AI systems for fracture detection in clinically relevant musculoskeletal imaging settings and reported diagnostic or workflow outcomes informative for clinical interpretation or implementation. Eligible designs included systematic reviews, meta-analyses, multi-reader multi-case observer studies, retrospective and prospective cohort studies, and real-world implementation studies. Priority was given to studies comparing AI-assisted human interpretation with unaided human reading, assessing stand-alone AI performance against human readers or reference standards, or examining deployment-related outcomes in emergency, trauma, or orthopedic imaging. Studies focused exclusively on technical model development without clinically interpretable diagnostic outcomes, non-fracture tasks, non-musculoskeletal applications, duplicate publications, and conference abstracts lacking sufficient methodological or outcome data were excluded from the review. Titles and abstracts were screened for relevance by W.M.G., with independent verification by R.O. and P.K. Full-text articles considered potentially eligible were assessed against the predefined inclusion criteria. Any uncertainties regarding study eligibility were resolved through discussion and consensus. Consistent with the structured narrative design, the study selection process prioritized clinical relevance, methodological informativeness, and applicability to real-world fracture diagnoses.

### 2.4. Data Extraction

Data extraction was performed using a predefined framework. The extracted variables included study design, clinical setting, anatomical region, imaging modality, AI system characteristics, mode of AI use (stand-alone versus assistive), reader characteristics and level of experience, reference standard, diagnostic performance measures, and selected workflow-related outcomes. The diagnostic performance variables included sensitivity, specificity, area under the receiver operating characteristic curve (AUC), missed fracture rates, interpretation time, and inter-reader agreement. Initial data extraction was performed by W.M.G. and independently verified by R.O. and P.K. Any discrepancies were resolved through discussion and consensus among the authors. Reported values were extracted as published; no recalculation, statistical reweighting, or de novo meta-analytic synthesis of primary data was performed.

### 2.5. Qualitative Assessment of Bias and Heterogeneity

A formal study-level quality scoring tool was not applied because the included evidence comprised heterogeneous study designs, including meta-analyses, multi-reader observer studies, retrospective and prospective cohort studies, and real-world implementation studies. Instead, potential sources of bias and methodological limitations were qualitatively considered during the synthesis, informed by established frameworks for diagnostic accuracy studies, including QUADAS-2 and emerging AI-specific reporting guidance [42,43]. Particular attention was paid to selection bias, spectrum bias, verification bias, enriched fracture prevalence in observer studies, heterogeneity of reference standards, and differences between stand-alone algorithm evaluation and clinically embedded reader-assisted studies. Additional attention was given to the radiograph-versus-CT reference-standard mismatch in spinal fracture studies, as this may affect the interpretability of performance estimates derived from radiograph-based models trained or validated against CT-confirmed outcomes. These considerations informed the interpretation of the apparent diagnostic gains and the formulation of conclusions regarding anatomy-specific implementation.

### 2.6. Structured Narrative Synthesis and Table Construction

Because of substantial heterogeneity across anatomical domains, study designs, AI deployment modes, reader expertise, and reported outcomes, no pooled effect estimates, meta-regression analyses, or indirect comparative rankings of individual AI systems were performed. Instead, the findings were synthesized narratively across prespecified clinically relevant domains. The synthesis was organized along three primary axes:

(1) The overall diagnostic impact of AI assistance compared with unaided human readers and stand-alone AI systems; (2) reader-experience-dependent effects of AI support; and (3) anatomy-specific patterns of diagnostic benefit, uncertainty, and potential clinical risk.

The diagnostic performance findings were summarized using reported ranges and directional patterns rather than pooled summary estimates. The quantitative values presented in the text and tables were intended to reflect published findings from representative studies and reviews, not meta-analytic summary measures generated de novo by the authors. For anatomy-specific summary tables, the consistency of evidence was qualitatively assessed based on concordance across study designs, the direction of observed effects, and the breadth of supporting evidence. “High consistency” indicated broadly concordant findings across multiple studies or reviews; “moderate consistency” indicated generally similar directional findings with some heterogeneity; and “low consistency” indicates sparse, conflicting, or methodologically limited evidence.

Qualitative labels such as “high”, “moderate”, “variable”, or “moderate to strong” were used as interpretive synthesis descriptors based on the direction, consistency, and approximate magnitude of reported effects across the included studies; they should not be interpreted as formal evidence grades or standardized quantitative categories.

This synthesis approach has inherent methodological constraints. As a structured narrative review, it does not provide the same level of reproducibility as a formally registered systematic review with exhaustive database-specific search updating and formal study-level risk-of-bias scoring. In addition, the underlying literature is methodologically heterogeneous, which limits direct comparability across anatomical regions, clinical settings, and study designs. Some of the included evidence also derives from retrospective observer studies or enriched test sets that may not fully reflect real-world fracture prevalence or workflow conditions. Accordingly, the present synthesis was designed to prioritize clinical interpretability, implementation relevance, and diagnostic safety over formal quantitative aggregation. This approach is consistent with contemporary guidance on narrative evidence synthesis in complex and heterogeneous studies, which emphasizes the explicit justification of the review design and the transparent reporting of analytic procedures [44].

## 3. Results

### 3.1. Evidence Base and Study Landscape

The included literature comprised a heterogeneous yet clinically informative evidence base, including systematic reviews and meta-analyses of diagnostic test accuracy, multi-reader and multi-case (MRMC) observer studies, retrospective and prospective cohort studies, and real-world emergency department (ED) implementation studies. Across these investigations, AI systems were evaluated both as stand-alone diagnostic models and, more commonly, as assistive tools integrated into human image interpretation workflows. The evidence covered multiple anatomical regions, with the greatest number of studies involving hip fractures, wrist and hand fractures, and broader appendicular skeletal injuries. The study sizes ranged from small observer series to large retrospective datasets, including hundreds of thousands of radiographs, with reader panels typically ranging from a few to several dozen clinicians. The representative studies included in the structured narrative synthesis are summarized in Supplementary Table S1.

Across study designs, a consistent pattern emerged: the most clinically relevant and reproducible benefit of AI was observed when the technology was used to support human readers, rather than replacing them. Simultaneously, the magnitude and reliability of the diagnostic benefit varied by anatomical region, reader experience, reference standard, and study design, with substantial differences across these factors.

### 3.2. Overall Diagnostic Performance: Stand-Alone AI, Human Readers, and AI-Assisted Interpretation

Across recent meta-analyses and large observer studies, stand-alone AI systems generally demonstrated high diagnostic accuracy for fracture detection on radiographs, with reported sensitivities and specificities typically in the high-80% to low-90% range, and AUC values frequently exceeding 0.90 [11,21,31,32,45]. However, the stand-alone AI performance was not consistently superior to that of experienced radiologists or musculoskeletal imaging specialists, particularly in anatomically challenging regions or where fracture morphology was subtle, complex, or heterogeneous [11,33,37,38,39,46].

In contrast, AI-assisted human interpretation showed a more consistent and clinically relevant pattern of benefit. Across reviews and MRMC studies, AI support most commonly improved sensitivity, whereas specificity was usually preserved or changed only minimally [29,35,47,48,49]. In representative studies, AI assistance was commonly associated with moderate sensitivity gains and lower missed-fracture rates compared with unaided human interpretation [33,34,49,50,51]. These findings support the interpretation of AI as a diagnostic safety net that helps reduce perceptual oversight during the initial image assessment rather than as a substitute for expert clinical judgment [33,48,51,52]. The overall diagnostic performance patterns are summarized in Table 1. As outlined in Section 2.6, these values are presented as directional summaries of the published findings rather than as de novo pooled estimates.

### 3.3. Reader-Experience-Dependent Effects

The magnitude of the AI benefit was not uniform across the reader groups. Across multi-reader and subgroup analyses, less-experienced readers—including trainees, junior doctors, emergency physicians, and other nonspecialist clinicians—generally showed the greatest absolute gains in diagnostic performance with AI assistance [33,34,51,60,61]. In these settings, AI support most often improved sensitivity and reduced missed fractures, thereby narrowing, although not eliminating, the gap between inexperienced and expert readers [25,34,48,58,62].

In contrast, the incremental diagnostic benefit of AI was smaller among experienced radiologists and musculoskeletal imaging specialists. In expert readers, the contribution of AI was more often reflected in improved detection of subtle injuries, reduced interpretive variability, and, in some studies, shorter reading time rather than large gains in baseline diagnostic accuracy [33,53,63]. Across studies, the largest gains were generally reported in less-experienced reader groups [33,34,53]. The cross-study patterns by reader category are summarized in Table 2.

### 3.4. Anatomical Region-Specific Diagnostic Performance

The diagnostic performance varied substantially across anatomical regions, and this variation was one of the findings of this review. The most consistent benefits of AI assistance were observed in appendicular skeletal imaging, including wrist, hand, ankle, elbow, knee, and long bone fractures. In these domains, AI-assisted readers generally achieve improved sensitivity with little or no major loss of specificity. In contrast, the performance for rib and spinal fractures was less favorable and more variable, with smaller or inconsistent gains in sensitivity, greater loss of specificity, and more interpretive noise, underscoring the need for greater caution in these regions [24,35,68,71,72,73,74].

Across studies, AI-assisted detection of hip and proximal femur fractures showed consistently strong diagnostic performance. Across studies, reported sensitivities and specificities for hip fracture detection were typically in the high-80% to low-90% range, with AUCs around 0.91–0.99, and several reports suggested that AI support could help less-experienced readers approach the performance of orthopedic or musculoskeletal imaging specialists [54,75,76,77]. However, occult or minimally displaced hip fractures remain an important limitation, and escalation of imaging may still be required in selected cases.

Wrist and hand fractures also showed strong performance, although with greater variability than hip fractures. AI assistance is particularly useful for detecting subtle or minimally displaced injuries. However, in some studies, this benefit was accompanied by an increased rate of false-positive prompts, especially in clinically ambiguous cases [68,78,79,80].

Rib fractures exhibited a more mixed pattern. Several studies have suggested that AI may improve sensitivity in emergency and after-hours settings; however, this benefit is sometimes offset by reduced specificity and increased interpretive noise [24,52,64,74].

In contrast, axial skeletal fracture detection was less reliable. The weakest performance was reported in radiograph-based detection of cervical spine trauma and vertebral fractures, where AI sensitivity was lower and less consistent than that of attending radiologists in representative studies [37,38,67]. Thoracic and lumbar vertebral fracture tasks also showed variable performance, indicating that spinal fracture detection should not be treated as a single homogeneous category. Across studies, spinal fracture detection showed lower and less consistent performance than hip or appendicular radiography. The anatomy-specific benefit–risk profiles of AI-assisted fracture detection are summarized in Table 3.

### 3.5. Subtle and Occult Fractures: Diagnostic Gains and Trade-Offs

AI assistance was reported to be valuable in detecting subtle, non-obvious, and often overlooked fractures. Across studies, the relative diagnostic benefit of AI was often greatest for minimally displaced injuries, avulsion fractures, and other lesions prone to perceptual oversight during routine initial reading, with typical sensitivity gains of approximately 10–15 percentage points reported in these subgroups [65,83,84].

This pattern was also observed in the selected task-specific studies. For example, in a multicenter study of extremity radiographs, lesion-level detection of avulsion fractures was higher with the optimized AI model than that of radiologists (57.9% vs. 29.8%). In contrast, no significant difference was observed between non-avulsion fractures [85].

However, these gains were not without cost. Improved sensitivity to subtle findings is frequently accompanied by an increase in false-positive suggestions, particularly in the wrist, scaphoid, and other anatomically complex or low-signal contexts [24,70,86].

The benefit–risk balance across subtle and occult fracture patterns is presented in Table 4.

### 3.6. Workflow and Efficiency Outcomes

In addition to diagnostic accuracy, several studies have reported the workflow-related effects of AI assistance. Across observer and early implementation studies, AI support was associated with reductions in image interpretation time and improvements in inter-reader agreement. However, the magnitude of these effects varies substantially across anatomical regions and study designs [33,51,69,87].

Importantly, these efficiency gains were generally observed without major reductions in diagnostic accuracy in the reported studies. The available evidence remains heterogeneous, and the clinical relevance of faster interpretation depends on the balance between speed and diagnostic safety [10,51,71]. Several studies reported shorter interpretation times and, in some cases, improved inter-reader agreement when AI was used within supervised human workflows [59,66].

## 4. Discussion

### 4.1. Principal Findings

This review suggests that the clearest and most reliable clinical value of AI-assisted fracture detection lies in supporting human readers, rather than replacing them. Across a heterogeneous body of evidence, the most consistent and clinically relevant benefit was a reduction in missed fractures at the initial imaging assessment [10,51,57,71]. This pattern was especially evident in radiograph-based workflows and among less-experienced readers working in busy orthopedic, trauma, or emergency settings [35,61].

Simultaneously, the effect of AI assistance is context-dependent. Anatomical region and reader experience emerged as the main factors shaping both benefits and risks [13,24,34,39,51,60,88,89]. Therefore, the practical question is not whether AI works for fracture detection in general, but where it performs well enough to justify implementation, where the gains are modest, and where the limitations may introduce unacceptable diagnostic risk [51,57,71,90]. This anatomy- and context-specific variation is the central message of this review.

### 4.2. AI as a Diagnostic Safety Net Rather than an Autonomous Reader

A recurring finding across reviews, MRMC observer studies, and early implementation reports is that AI assistance tends to improve fracture detection primarily by increasing sensitivity while maintaining acceptable specificity [10,11]. This was most apparent in readers with limited experience in musculoskeletal imaging. In practical terms, AI seems to function best as a second reader or structured decision-support tool that helps reduce perceptual oversight and supports a more systematic review of radiographs under time pressure [11,35,51].

In contrast, stand-alone AI performance, although often strong on selected tasks, is generally comparable to, rather than consistently better than, expert radiologists or musculoskeletal imaging specialists [11,24,32,69]. Therefore, the real diagnostic value comes from human–AI collaboration rather than autonomous decision-making [51,71,90]. This has direct implications for deployment strategy, workflow design, and medico-legal responsibility and is broadly consistent with current regulatory and ethical expectations for clinical AI [71,91,92,93].

### 4.3. Reader Experience Modifies the Clinical Value of AI

One of the most clinically important findings of this review is that the benefit of AI is strongly influenced by reader experience. Less-experienced readers, including trainees, junior doctors, emergency physicians, and other non-specialists, generally derive the greatest benefit, most often through higher sensitivity and fewer missed fractures [26,35,55]. In practical terms, AI helps narrow the gap between inexperienced and expert readers, even if it does not completely close that gap [34,35,51,53,55].

Among experienced radiologists and musculoskeletal imaging specialists, the incremental benefit is usually smaller and more selective. In these settings, AI may still add value by reducing interpretive variability, improving vigilance for subtle findings, or improving efficiency; however, it is less likely to produce large absolute gains in baseline diagnostic accuracy [45,49,51,57]. This pattern supports a selective implementation strategy in which the rationale for AI use varies across institutions and reader groups, with the strongest case in high-volume environments staffed predominantly by less-experienced readers [24,94,95].

This experience-dependent pattern extends to pediatric practices. A recent systematic review and meta-analysis focusing on pediatric fracture detection on radiographs reported a pooled sensitivity of 93% (95% CI 92–94%), specificity of 91% (95% CI 88–93%), and an AUC of 0.96 (95% CI 0.92–0.97), with performance maintained in external validation cohorts [28]. At the same time, the authors highlighted substantial heterogeneity in anatomical coverage, case mix, and reader experience. They noted that many contributing datasets were single-center and retrospectively enriched, which may limit the direct generalizability of these pooled estimates across all emergency departments [28,96].

### 4.4. Anatomical Region as the Primary Factor Determining Benefits and Risks

The most important factor influencing the utility of AI for fracture diagnosis is the anatomical region under examination. Available evidence generally indicates a favorable benefit-to-risk ratio for skeletal imaging of the extremities and the detection of proximal femoral fractures [24,32,46,51,54,97]. In these anatomical regions, AI support most consistently improves sensitivity, reduces the number of missed fractures, and appears suitable for supervised routine use in trauma and emergency imaging [10,35,40,46,69,79]. Currently, these are the most mature and clinically justified applications of AI-assisted fracture detection [11,24,51,54].

However, anatomical regions differ not only in their inherent diagnostic difficulty but also in the clinical consequences of error [14,20,65]. In some situations, a moderate increase in false-positive suggestions may be acceptable if the primary priority is reducing missed injuries. In other cases, especially where both the false reassurance of no injury and unnecessary escalation have serious consequences, the same AI behavior may be far less acceptable. Rib fractures occupy an intermediate position, where increased sensitivity is often offset by reduced specificity and greater interpretive noise [52,64,74]. Cervical and thoracolumbar spine injuries remain a high-risk area where current AI based on radiographs shows limited and inconsistent benefits compared to expert radiologists [38,71,98,99].

Similar difficulties are observed in cases of complex, multi-site, comminuted, displaced fractures. In a large-scale evaluation of commercial algorithms in real-world settings, performance for acute single fractures remained within the expected range, but dropped significantly in studies involving multiple fractures, highlighting the risk of overestimating AI reliability in polytrauma scenarios [24,39,51,59,65]. Similarly, although overall fracture detection accuracy in children is generally high, lesion-level sensitivity may still be lower for certain subtle injury subtypes, including specific avulsion fracture patterns [28,36,95,96]. In summary, the results reported in the literature suggest that AI is currently most reliable for typical tasks involving single fractures [11,24,33,51], whereas rare, multi-site, or difficult-to-diagnose injury patterns remain higher-risk applications for AI, requiring close expert supervision [36,39,59,98].

Accordingly, implementation decisions should be guided not only by overall accuracy metrics but also by anatomy-specific risk tolerance and the clinical consequences of missed or overcalled injuries.

### 4.5. Why Spinal Fracture AI Remains Problematic

The weakest and most clinically concerning performance pattern identified in this review involved spinal fracture detection, particularly radiograph-based assessments of traumatic cervical spine injuries. This should not be viewed solely as a governance or deployment issue. More fundamentally, it likely reflects structural imaging limitations, fracture heterogeneity, and a mismatch with reference standards, all of which make spinal fracture detection intrinsically more difficult for current AI systems than hip or appendicular fracture tasks [17,38,73,98,100,101,102].

Compared with appendicular radiography, cervical spine radiography is more challenging because of anatomical overlap, incomplete visualization, projection-dependent ambiguity, and the possibility that clinically important fractures may be occult or only subtly visible on plain films [73,100,103,104,105]. These features complicate both human interpretation and AI-based detection. In addition, spinal fractures are morphologically heterogeneous, spanning traumatic cervical injuries, thoracolumbar trauma, and osteoporotic vertebral compression fractures [71,81,99,103,106,107]. These tasks are not interchangeable and should not be treated as a single category.

Reference standard mismatch is another major issue. Many spinal fractures, especially in the cervical spine, are confirmed on CT despite being only subtly visible or not confidently visible on radiographs [3,103,105]. When radiograph-based AI systems are trained or validated against CT-confirmed endpoints, the target may be clinically relevant but methodologically problematic, as the model is effectively asked to infer findings that may not be fully resolvable from the input modality [108,109,110]. This likely contributes to the lower and less reliable performance of spinal fracture AI compared with hip and appendicular applications. For these reasons, traumatic cervical spine fracture detection on radiographs should still be regarded as a high-risk, lower-maturity use case that requires substantial caution. Vertebral fracture detection, including osteoporotic compression fractures, should be considered a related but distinct task with different imaging characteristics, clinical aims, and implementation implications [24,71,81,82].

### 4.6. Subtle Fractures: Benefit and Diagnostic Noise

AI assistance is particularly advantageous for detecting subtle, minimally displaced, and often overlooked fractures [35,51]. In these cases, its role is not to confirm obvious findings but to heighten awareness of abnormalities that might otherwise be missed during routine evaluations. AI-assisted fracture detection may be particularly helpful for subtle injuries that are at higher risk of being overlooked, but gains in sensitivity can also introduce interpretive noise and false-positive alerts. This tendency appears more pronounced in anatomically complex regions, in the presence of normal variants, and in workflows with limited opportunities for downstream adjudication. In such settings, AI support for subtle fractures is best positioned as a structured second reader, with routine human verification and clinical correlation, rather than as a signal to be accepted uncritically [21,24,26,29,47,51,111]. Table 4 provides a summary of the typical benefit–risk balance across major, subtle, and occult fracture patterns.

### 4.7. Clinical Governance and Implementation Considerations

The findings of this review support selective and supervised clinical implementation. In practical terms, safe implementation requires local validation, anatomy-specific deployment criteria, explicit clinician accountability for the final report, and post-deployment monitoring for performance drift or unintended workflow effects [112,113,114].

These safeguards are especially important because the clinical value of AI depends on more than model performance alone. It is also shaped by reader expertise, case mix, disease prevalence, and how the tool is integrated into reporting workflows [33,35,39,59,94]. Consequently, the same AI system may have very different practical value in a specialized trauma center, a general emergency department, or a lower-volume orthopedic service [33,35,39,59,94,112].

Patient-facing considerations, including transparency of data use, trust, and governance of image transfer or cloud-based infrastructure, are also relevant to implementation. However, because these issues were not systematically addressed across the included studies, they should be regarded as important contextual considerations rather than as conclusions directly established by the present evidence base [112,113,114,115,116,117].

Available evidence also suggests potential efficiency gains in selected high-volume settings. Still, current data remain insufficient to support strong economic conclusions [56,117], and diagnostic improvements should not be assumed to automatically translate into better patient outcomes [14,45,56,71].

### 4.8. Limitations of This Review

This review should be interpreted in light of several methodological limitations. First, as a structured narrative review, it does not provide the same degree of reproducibility as a formally documented systematic review, which includes exhaustive search updates, multiple verifications at all stages, and a formal assessment of the risk of bias at the individual study level. Therefore, the selection and synthesis of studies inevitably involved some degree of subjective judgment by the authors, which raises the possibility of selection bias and subjective interpretation.

Second, the evidence on which this review is based is heterogeneous in several important respects. The included literature comprises systematic reviews, multi-observer studies, retrospective and prospective cohort studies, and early implementation studies, which differ significantly in anatomical scope, case diversity, readers’ expertise, AI implementation mode, and reference standards. This heterogeneity limits direct comparability across anatomical regions and clinical settings, diminishing the strength of any uniform cross-domain conclusions. The included evidence also spans different stages of technological and translational maturity, which further limits the stability of broad cross-study conclusions about current clinical usefulness.

Third, a significant portion of the available evidence remains dominated by retrospective studies and enriched datasets, in which fracture incidence and case composition may differ significantly from routine practice in emergency or trauma care. This may overestimate apparent diagnostic performance and limit the generalizability of reported benefits to real-world practice. Furthermore, differences in reference standards across studies introduce additional uncertainty in interpretation, particularly for anatomically challenging tasks such as detecting spinal fractures.

Fourth, the quantitative ranges presented in this review should be considered a descriptive and indicative summary of the included literature, rather than precise comparative estimates. Given the heterogeneity of study designs, anatomical tasks, reference standards, reader groups, and reported outcomes, direct quantitative comparisons between studies remain limited. These limitations do not negate the value of the review but require cautious interpretation. This synthesis aimed to identify clinically meaningful patterns regarding diagnostic benefits, risks, and implementation implications, rather than to create formal comparative rankings of AI systems or anatomy-specific quantitative summary measures.

Finally, the review was limited to English-language studies published between January 1, 2021, and March 31, 2026, focusing primarily on fracture detection based on X-ray images. Although this scope was deliberately chosen to address the main clinical question regarding AI as a second-reader tool during the initial assessment of trauma, it may have excluded earlier studies, studies in languages other than English, or studies not based on X-ray images, which could have provided additional contextual perspective.

## 5. Future Research

Future studies should not focus solely on reconfirming the high diagnostic accuracy of AI, but rather on its practical application in clinical practice, its limitations across anatomical areas, and its impact on patient outcomes.

First, prospective, realistic studies are needed to determine whether the use of AI actually improves diagnostic safety, workflow, and follow-up care for patients. Results obtained in retrospective studies do not always translate into real clinical benefits.

Second, detecting spinal fractures requires more precise studies. A clear distinction must be made between the detection of post-traumatic cervical spine fractures and the diagnosis of vertebral body fractures, such as osteoporotic fractures, as these are different diagnostic challenges. Better-suited datasets, clear task definitions, and robust reference standards are necessary.

Third, future studies should more closely examine how AI performance depends on the clinician’s experience, the types of cases, and the work environment. The greatest benefits may be seen when working with clinicians in training, non-specialists, or in high-workload settings such as emergency departments—but this requires confirmation in prospective studies.

Fourth, greater attention should be paid to approaches that combine different data sources, particularly in diagnostically challenging cases, such as spinal injuries. Combining data from X-rays and computed tomography or integrating imaging data with clinical information can help overcome the limitations of individual methods.

Finally, the evaluation of AI implementation should consider not only the algorithm’s accuracy but also implementation costs and challenges, oversight requirements, and the actual impact on clinical practice.

## 6. Conclusions

AI systems for fracture detection are of greatest clinical value when they support the physician rather than replace their judgment. Available studies show that the greatest benefit lies in reducing the number of missed fractures on X-rays, particularly in high-workload settings and among less-experienced physicians.

AI performance varies across anatomical regions. The best and most consistent results are seen in detecting proximal femur and limb fractures, where AI improves diagnostic sensitivity without compromising overall accuracy. For spinal fractures, the situation is more complex—current systems are less effective, mainly due to imaging challenges, the wide variety of fractures, and differences between X-ray and computed tomography images. These limitations stem from the very nature of the problem, not just from implementation issues, which define the current boundaries of AI applications in this field.

The physician’s experience is also significant. AI better supports less-experienced users, whereas for experts, its role is more auxiliary—it aids in detecting subtle changes, increases vigilance, and helps maintain consistency in assessment, but does not lead to a significant improvement in basic diagnostic accuracy.

In summary, the available data indicate that AI should be implemented selectively, depending on the anatomical area, and always under clinical supervision. The key issue is not whether to use AI everywhere, but where it actually improves diagnostic safety in a repeatable and clinically acceptable manner. Currently, the most justified model is the use of AI as a “second reader” or decision-support tool, operating within the framework of responsible interpretation by a physician.

## Figures and Tables

**Table 1:** Qualitative summary of overall diagnostic performance patterns in AI-assisted fracture detection.

Outcome Domain	Typical Reported Pattern	Main Clinical Interpretation	Evidence Base	Supporting References
Sensitivity	Usually improved with AI assistance; gains were commonly moderate to large in reader-assistance settings	AI most often functions as a diagnostic safety net by reducing perceptual oversight during initial image assessment	Meta-analyses; MRMC observer studies; selected implementation studies	[10,11,24,33,34,35,51,53]
Specificity	Usually preserved or only slightly affected	Sensitivity gains were often achieved without major loss of specificity, although this varied by anatomical region and task complexity	Meta-analyses; MRMC observer studies	[10,11,24,30,32,33,40,51]
Stand-alone AI discrimination	Often high, but not consistently superior to expert readers across all anatomical tasks	Stand-alone AI performance may approximate expert-level accuracy in selected tasks, but remains less reliable in anatomically complex or heterogeneous settings	Meta-analyses; stand-alone diagnostic accuracy studies	[11,21,24,31,32,45,54]
Missed fracture rate	Often reduced when AI is used as an assistive second-reader tool	The most clinically relevant benefit of AI assistance is reduction of overlooked fractures rather than replacement of expert judgment	MRMC studies; ED implementation studies	[10,26,33,35,40,47,49,51,53,55]
Reading time and workflow efficiency	Frequently shortened in observer and implementation studies, although effects vary by workflow and anatomical task	Potential efficiency benefits appear most relevant when AI is integrated into supervised human interpretation workflows	Implementation studies; observer studies; workflow analyses	[10,39,49,56,57,58,59]

Footnote: Reported patterns summarize the direction and approximate clinical magnitude of findings across recent meta-analyses, multi-reader multi-case (MRMC) observer studies, and real-world implementation reports. No quantitative pooling, reweighting, or reanalysis of primary data was performed. The table is intended to provide a qualitative overview of cross-study tendencies rather than precise comparative summary estimates.

**Table 2:** Reader-experience-dependent effects of AI assistance in fracture detection.

Reader Category	Baseline Diagnostic Performance	Effect of AI Assistance	Typical Clinical Interpretation	Consistency Across Studies	Supporting References
Emergency physicians	Moderate sensitivity; high variability	Large sensitivity gain; substantial reduction in missed fractures	Strong safety-net effect in high-volume ED and acute-care settings	High	[26,33,49,55,58,64,65]
Junior radiology residents	Moderate sensitivity	Large sensitivity gain; improved detection of subtle errors	Compensation for limited experience; support for standardization	High	[10,26,40,47,53,55,66]
General radiologists	High sensitivity	Small to moderate improvement	Reduction in overlooked subtle findings; modest workflow support	Moderate	[10,11,33,35,49,67,68]
Musculoskeletal radiologists	Very high sensitivity	Minimal absolute gain; occasional subtle benefits	Main value is efficiency and detection of rare or easily missed lesions	Moderate	[10,35,47,67,69,70]

Footnote: Reader categories and effect magnitudes were synthesized qualitatively from subgroup analyses and comparative MRMC observer studies. The table reflects the direction and consistency of reported findings rather than pooled quantitative estimates.

**Table 3:** Qualitative synthesis of anatomy-specific patterns in AI-assisted fracture detection.

Anatomical Region	Stand-Alone AI Performance (Qualitative)	AI-Assisted Reader Performance (Qualitative)	Predominant Reported Benefit	Key Risks/Limitations	Evidence Consistency	Supporting References
Hip/proximal femur	High	Very high	Marked sensitivity gains and fewer missed fractures on radiographs	Occult or minimally displaced fractures may still require escalation of imaging in selected cases	High	[54,75,76,77]
Appendicular skeleton (general)	High	High	Consistent sensitivity gains across long bones and extremities	Performance varies across AI systems and specific fracture subtypes	High	[24,33,35,40,66]
Wrist/hand	Moderate to high	High	Improved detection of subtle and minimally displaced fractures	Increased false-positive prompts and greater interpretive noise in complex cases	Moderate	[68,78,79,80]
Rib fractures	Moderate	Moderate to high	Improved sensitivity in emergency and after-hours settings	Reduced specificity and possible downstream escalation to additional imaging, including CT in selected cases	Moderate	[24,52,64,74]
Cervical spine trauma on radiographs	Low	Low to moderate	Limited or selective benefit in some settings	Anatomical overlap, incomplete visualization, CT-reference mismatch, and high-risk false negatives	Low	[37,38,67,73]
Thoracic/lumbar vertebral fracture tasks	Low to moderate	Variable	Possible support in selected vertebral fracture tasks	Morphological heterogeneity, inconsistent performance, and non-equivalence to cervical trauma detection	Low to moderate	[24,71,73,81,82]

Footnote: The categories shown in the table are qualitative synthesis labels assigned by the authors based on concordance across reviews, observer studies, and implementation reports. They summarize the predominant patterns in the literature and do not constitute formal evidence grading or comparative ranking of individual AI systems.

**Table 4:** Qualitative synthesis of benefit–risk patterns in AI assistance for subtle and commonly overlooked fractures.

Fracture Pattern	Typical AI-Related Gain	Main Diagnostic Benefit	Main Trade-Off	Suggested Clinical Safeguard	Supporting References
Minimally displaced fractures	Moderate	Improved detection of subtle cortical disruption	Increased interpretive noise and false positives	Human adjudication and local threshold calibration, where applicable	[65,83,84]
Avulsion fractures	Moderate to strong	Better lesion-level sensitivity for small osseous fragments	Overcalling anatomical variants or artifacts	Correlation with anatomy and clinical findings	[65,84,86]
Occult or initially overlooked fractures	Positive but variable	Increased vigilance during first-pass reading	More false-positive prompts and follow-up imaging	Structured second-reader workflow and escalation imaging when clinically indicated	[21,24,51,65,86]

Footnote: Patterns and effect magnitudes were qualitatively synthesized from subgroup analyses and task-specific studies focusing on subtle and occult fractures. The reported gains and trade-offs illustrate typical tendencies rather than pooled quantitative estimates. Qualitative gain descriptors reflect interpretive judgments of synthesis rather than standardized effect-size categories.

## Data Availability

No new data were created or analyzed in this study. Data sharing does not apply to this article.

## Supplementary Materials

The following supporting information can be downloaded at: https://www.mdpi.com/article/10.3390/diagnostics16101420/s1, Supplementary Table S1.
