# Supplementary material for: AI-Assisted Fracture Detection in Orthopedic and Trauma Imaging: Where It Works, Where It Fails, and Principles for Safe Clinical Deployment
Source: Diagnostics (Basel). 2026 May 7;16(10):1420. doi: 10.3390/diagnostics16101420 (PMC13206237; doi:10.3390/diagnostics16101420)
Supplement: Supplementary file 1 [file diagnostics-16-01420-s001.zip › diagnostics-4196291-supplementary.pdf]

Supplementary Table S1

Database / purpose	Search field / filter	Search string / query (as used)
PubMed – exploratory core search	Title/Abstract; 2021–31 March 2026; English	"artificial intelligence"[Title/Abstract] AND fracture[Title/Abstract] AND (x-ray[Title/Abstract] OR radiograph[Title/Abstract] OR radiographs[Title/Abstract])
PubMed – clinically focused search (diagnostic accuracy & implementation)	Title/Abstract; 2021–31 March 2026; English	("artificial intelligence"[Title/Abstract] OR "machine learning"[Title/Abstract] OR "deep learning"[Title/Abstract]) AND (fracture*[Title/Abstract] OR "fracture detection"[Title/Abstract]) AND (radiograph*[Title/Abstract] OR "plain radiograph*"[Title/Abstract] OR "plain film*"[Title/Abstract] OR "x-ray"[Title/Abstract]) AND ("diagnostic accuracy"[Title/Abstract] OR sensitivity[Title/Abstract] OR specificity[Title/Abstract] OR observer[Title/Abstract] OR multireader[Title/Abstract] OR "reader performance"[Title/Abstract] OR implementation[Title/Abstract] OR workflow[Title/Abstract] OR "decision support"[Title/Abstract] OR "second reader"[Title/Abstract])
Web of Science Core Collection – clinically focused search	Topic (TS); 2021–2026; English; Article/Review	TS=(("artificial intelligence" OR "machine learning" OR "deep learning") AND (fracture OR "fracture detection") AND (radiograph OR radiographs OR "plain radiograph" OR "plain radiographs" OR "x-ray") AND ("diagnostic accuracy" OR sensitivity OR specificity OR observer OR multireader OR "reader performance" OR implementation OR workflow OR "decision support" OR "second reader"))
Scopus – clinically focused search	TITLE-ABS-KEY; 2021–2026; English; Article/Review	TITLE-ABS-KEY(("artificial intelligence" OR "machine learning" OR "deep learning") AND (fracture OR "fracture detection") AND (radiograph OR radiographs OR "plain radiograph" OR "plain radiographs" OR "x-ray") AND ("diagnostic accuracy" OR sensitivity OR specificity OR

Database / purpose	Search field / filter	Search string / query (as used)
		observer OR multireader OR "reader performance" OR implementation OR workflow OR "decision support" OR "second reader")) AND PUBYEAR > 2020 AND PUBYEAR < 2027
ScienceDirect – clinically focused search	All fields; 2021–2026; English; Research articles & reviews	"artificial intelligence" AND fracture AND (x-ray OR radiograph OR radiographs) AND detection AND ("diagnostic accuracy" OR "diagnostic outcomes")
Google Scholar – supplementary query 1	All fields; ≥2021	"artificial intelligence" fracture detection radiographs diagnostic accuracy
Google Scholar – supplementary query 2	All fields; ≥2021	"deep learning" fracture detection radiographs reader performance
Google Scholar – supplementary query 3	All fields; ≥2021	"AI-assisted" fracture detection radiographs implementation emergency department

Searches were restricted to articles published from 1 January 2021 to 31 March 2026. In Web of Science, Scopus, and ScienceDirect, only English-language articles and reviews were retained. Google Scholar was used only for supplementary retrieval, citation chasing, and identification of recent or ahead-of-print articles; results were manually screened to retain studies reporting fracture-level diagnostic outcomes on plain radiographs.
